# Policy Actors’ Perceptions of Conflicts of Interest and Alcohol Industry Engagement in UK Policy Processes

**DOI:** 10.34172/ijhpm.2024.8068

**Published:** 2024-01-28

**Authors:** Katherine Severi, Benjamin Hawkins

**Affiliations:** ^1^Institute of Alcohol Studies, London, UK.; ^2^MRC Epidemiology Unit, University of Cambridge, Cambridge, UK.

**Keywords:** Alcohol Policy, Conflicts of Interest, Alcohol Industry

## Abstract

**Background:** Alcohol industry organisations occupy a prominent position in UK alcohol policy, but their involvement has been contested by public health bodies on the basis that a conflict of interest (COI) exists between their economic objectives and those of public health. There are ongoing debates in the research literature about how to conceptualise COI and mitigate this in health research and practise. However, less attention has been paid to these issues in relation to the alcohol industry specifically. This article explores similarities and differences in beliefs among alcohol policy actors regarding COI and the implications of engagement with the alcohol industry in the context of UK public health policy.

**Methods:** Semi-structured interviews with a range of policy actors (n=26) including medical professionals, parliamentarians, civil servants, academic researchers, health campaigners, and alcohol industry representatives. Interviews with alcohol industry representatives were supplemented with an analysis of industry responses to a public consultation. All data was thematically coded using NVivo software.

**Results:** Two competing "coalitions" were identified, expressing beliefs about COI linked to alcohol industry engagement. Both divergent and convergent beliefs were expressed by the two coalitions in relation to the type of industry actor, form of engagement, the policy issue under discussion and the stage of policy process.

**Conclusion:** Alcohol policy is a complex and contested space in which policy actors have differing, nuanced and contingent understandings of COI and identify varying risks associated with alcohol industry engagement. In identifying the areas of convergence and diversion in both understanding and evaluation of COI in alcohol-specific settings, these findings will assist both decision-makers and non-governmental actors in developing policies and guidelines to manage potential COI in future.

## Background

**Key Messages**

**Implications for policy makers**

- Conflicts of interest (COIs) relating to alcohol industry engagement in public health policy settings are identified by policy actors as a major barrier to reducing alcohol-related harm.
- However, the concept of COI is not well understood and is often imprecisely defined in this context.
- Policy actors’ beliefs about COI and alcohol industry involvement in public health are more nuanced than is presented in the scholarly literature and policy debates.
- Facilitating reflection on, and understanding of, COI relating to alcohol policy may lead to greater clarity in policy debates and more effective engagement between policy actors.
- Findings from this study may inform the development of guidelines to manage and protect alcohol policy from COI and deliver better public health outcomes.

**Implications for the public**

Alcohol industry interference has been identified as a major barrier to public policy progress to reduce alcohol harm. This article helps understand how different policy actors view alcohol industry involvement in policy, and their beliefs on what sorts of interaction are appropriate and not appropriate. Gaining a better understanding of how these people view the role of the alcohol industry will help to inform the development of guidelines to mitigate risks associated with commercial conflicts of interest (COIs). Such guidelines could limit industry interference in future and allow for the enactment of public health policies to save lives.

Alcohol policy debates are highly contentious and involve differing understandings of the nature of alcohol-related problems and the most appropriate policy responses. The international research consensus is that the most effective way to reduce alcohol harms are population-level interventions to increase the price, reduce the availability and restrict the promotion of alcohol products,^1,2^ which have been identified by the World Health Organization (WHO) as their policy “best buys.”^3^ While these “upstream” policy measures are supported by public health advocates, they are widely opposed by the global alcohol industry which favours instead targeted, individual-level measures and self-regulatory regimes for which there is limited supporting evidence.^4^ At the same time, industry actors question the extent of alcohol-related harms and/or attempt to focus debates on certain forms of harm such as heavy episodic (“binge”) drinking or the most harmful and hazardous drinkers.^4-7^ Whilst governments in Scotland and Wales have progressed with evidence-based measures such as minimum unit pricing (MUP), alcohol policies and strategies in England have consistently eschewed the most effective policy responses and have failed to achieve significant reductions in alcohol harms.^8^ Previous studies suggest this lack of effectiveness is due, at least in part, to alcohol industry influence over the policy process and their ability to shape the policy agenda towards industry-favoured but ineffective measures.^9^

 Public health actors have consistently argued that alcohol industry involvement in public health policy represents a conflict of interest (COI) because of private companies’ fiduciary responsibility to maximise shareholder value and thus to prioritise profits over public health goals.^10^ While there is little evidence of their effectiveness, the counter-measures favoured by alcohol industry actors are those least likely to affect their current industry business models and profits, since they do not look to reduce aggregate, population-level consumption and thus sales. As such, industry interests stand in direct opposition to those of public health.^11^ Despite this perceived COI, alcohol industry groups continue to occupy a prominent place in UK alcohol policy, enjoying significant access to policy-makers and participating in various self- and co-regulatory initiatives with government.^7,12^

 International guidelines exist to protect public health policy from commercial vested interests in relation to tobacco control, infant formula and foods high in fat, salt and sugar.^13,14^ However, there is no equivalent of these in the field of alcohol policy and there is little guidance available for policy-makers, non-governmental organisations (NGOs) and researchers to manage COI when engaging alcohol industry actors. The lack of established guidelines and principles means that, unlike in areas such as tobacco control,^13^ there is also no internationally agreed definition of COI in the context of alcohol. Furthermore, little is known about how different policy actors define the concept of COI, how they view alcohol industry engagement in the policy process and how this informs their professional practise.

 The research literature on the commercial determinants of health and elsewhere has engaged with the nature of COI and strategies to avoid or mitigate its effects. Akl and colleagues argue a COI exists “when a past, current, or expected interest creates a significant *risk* of inappropriately influencing an individual’s judgment, decision, or action when carrying out a specific duty” (emphasis added).^15^ Crucially, it is not necessary for an inappropriate judgment, decision, or action to have occurred, only that the potential for outside interests to affect these exists. The concept can be applied to various actors in government, policy-making and regulatory functions as well as researchers in receipt of external funding and civil society organisations. COI can relate both to individual as well as institutional interests and potentially also to those of close personal relationships to relevant actors. For example, if a spouse, partner or other relative of an individual responsible for key regulatory functions has a financial interest in that company, then a COI may be seen to exist. Strategies to identify and mitigate COI have focussed largely on transparency and disclosure mechanisms and, where the actual or perceived risk of a COI is seen to exist, recusal of actors from specific processes or decisions.^16,17^

 Further questions have arisen about what constitutes a relevant interest and whether the concept of COI should be limited to financial interests as in the preceding example or may be extended to other interest such as beliefs and ideologies. Some scholars have argued that there is no meaningful conceptual or practical distinction between financial and non-financial COIs and that individual characteristics such as religious denomination or political convictions should be treated in the same way, and subject to the same mitigation strategies as financial COI.^17-19^ Others, meanwhile, have argued that not all interests represent potential COIs and that extending the definition of COIs beyond financial interests runs the risk of undermining the concept and muddying the waters of how to manage more serious financial conflicts.^20^

 Previous studies have sought to capture the views of public health actors about the potential COI involved in working with health harming industries in comparative perspective (ie, tobacco, alcohol, and processed food),^21^ and with the alcohol industry specifically with a view to defining principles for engagement and non-engagement.^22^ These studies identify a high degree of consensus amongst respondents about the existence of COI, its application to specific industries and its consequences for professional practise. However, while cross industry studies have focussed specifically on the views of researchers, civil society actors and health advocates, those on the alcohol industry have focussed specifically on the research community. Similarly, the previous comparative study was international in scope and did not explore views of decision-makers. As such, there has been no study which has focussed on the views and beliefs of the full range of relevant actors on COI as it relates specifically to the alcohol industry.

 This article seeks to address this current gap in literature by exploring how alcohol policy actors understand the concept of COIs in relation to alcohol industry engagement in the context of UK public health policy. It begins from the premise that, to address problems associated with industry involvement in policy, a clearer understanding is required about what is meant by the term COI, as well as policy actors’ perceptions of, and beliefs about, this topic. It seeks also to identify the similarities and differences that exist within and between different groups of policy actors—defined as “belief coalitions”—on the issue of industry engagement and COI. In so doing, it aims to examine connections between respondents’ views on COI and the positions they hold on the nature of alcohol-related harms and the most effective policy responses.

## Methods

 A total of 26 semi-structured interviews were conducted with policy actors between January and September 2018.

###  Sampling and Recruitment

 Participants were purposively sampled based on their past or ongoing involvement in UK alcohol policy. A stakeholder analysis was conducted, identifying key alcohol policy networks and fora that were active between the years 2000-2018 inclusive. Participants were identified according to membership of the Public Health England Alcohol Leadership Board (the primary sample used to invite participants from civil service, health campaigning groups, medical and research backgrounds),^23^ the All-Party Parliamentary Group on Alcohol Harm (provided a sample of relevant Westminster parliamentarians)^24^ and the Portman Group Local Alcohol Partnership Network (provided sample of alcohol industry representatives).^25^ Six different groups of policy actors were identified: health campaigning groups, researchers, medical professionals, civil servants, Westminster (UK) politicians and alcohol industry representatives. Participants were presented with the list of six policy actor categories devised for this study and invited to self-identify with one of the groups for the purposes of reporting any material from their interview. In order to protect the anonymity of respondents in a small policy community, dealing with highly contentious topics, it is not possible to detail the specific positions occupied by actors beyond these sector descriptions. An overview of the recruitment of participants is provided in Table 1.

**Table 1:** Recruitment of Interview Participants

**Policy Actor Category**	**No. Invited to Interview**	**Accepted**	**No Response/Unable to Arrange Interview**	**Declined**	**Interview Conducted**
Civil servants	7	6	1	1	5
Health campaigners	6	5	1	0	5
Medical professionals	5	5	0	0	5
Academic researchers	5	5	0	0	5
Parliamentarians	6	6	2	0	4
Alcohol industry	13	6	4	7	2
Total	43	35	8	8	26

###  Data Collection

 Interviews were conducted face-to-face, except for three via Skype video call and lasted between 30 and 90 minutes. All interviews were voice recorded after securing written consent from participants and transcribed by the lead author. The data analysis framework was developed and refined during the study and is presented in Table 2.

**Table 2:** Data Analysis Framework

**Main Code**	**Sub-category**
Participant characteristic	Health campaigner
	Medical professional
	Academic researcher
	Civil servant
	Parliamentarian
	Alcohol industry representative
Understanding of alcohol harm	Harm increasing
	Harm decreasing
	Harm stabilising
	Drivers of harm
Alcohol Policy support	Pricing
	Availability
	Marketing
	Health service response
	Drink driving
	Education
	Industry partnerships
Understanding of COI	Financial
	Non-financial
	Actual
	Perceived
COI in alcohol policy settings	Existence of COI
	Evidence as a source of conflict
	Examples of industry obstructing policy
Alcohol Industry COI in policy settings	Type of actor
	Type of policy
	Stage of policy process
	Type of engagement
Suggestions for managing COI	Government motivations for working with industry
	Comparison to tobacco and food policy
	Experience of managing COI in professional setting

Abbreviation: COI, conflict of interest.

 The interview protocol was developed to guide interview questions, based on the findings from a narrative literature review, which informed the study’s scope and design. The framework was refined in an iterative process during data collection and subsequently formed the basis for developing the analytical framework.

###  Data Analysis

 Interviews were analysed in a two-step process. First, emergent themes that arose during the interviews themselves and during an initial review of the transcripts were identified and formed the basis of the analytical framework and codebook. Second, NVivo for Mac version 11.4.3 was used as a tool to analyse the interview data according to the final data analysis framework. Codes (termed “nodes” in NVivo) were set up corresponding with the “main code” and “subcategory” headings identified. Key arguments and emerging themes within each code were then identified and described by the lead author.^26^ The codebook was reviewed periodically and refined iteratively during the analysis process.

###  Documentary Analysis

 The relatively low number of alcohol industry representatives who were willing to participate in this study (n = 2) meant that, to ensure alcohol industry positions were more broadly represented, interviews were supplemented with a document analysis of alcohol industry submissions to the UK Health Select Committee inquiry into the UK Government’s 2012 Alcohol Strategy. This was the most recent opportunity for industry stakeholders to publicly express positions relating to the broad range of policy options available to reduce alcohol harm. Three alcohol industry submissions were identified for analysis reflecting the target of recruiting five respondents in each stakeholder category: the industry-funded charity Drinkaware,^27^ the industry self-regulatory body the Portman Group^28^ and multinational alcohol producer Diageo.^29^ The documentary analysis was performed after all interviews had been transcribed and coded and these organisations were selected on the basis of the frequency with which interviewees referred to them and their apparent importance in policy debates.

 Each document was coded using the same data analysis framework applied to interview transcripts. Information was available in each document for the codes “participant characteristic,” “understanding of alcohol harm,” and “alcohol policy support.” Little information was found in document text relating to COI, as this was not a focus of the chosen public consultation. The analysis therefore did not seek to code information pertaining to COI and was solely used to assess shared beliefs relating to alcohol harm and appropriate policy responses amongst members of the industry partnership coalition (IPC).

###  Theoretical Framework

 The aim of the study was to identify any connections between the positions adopted by policy actors on COI with their wider views about the health effects of alcohol and alcohol policy. To capture the commonalities that exist between different groups of actors we develop and apply the concept of “belief coalitions,” derived from the advocacy coalitions framework to describe groups of policy actors which adopt common, belief-oriented positions on a given policy issue, in this case alcohol-related harms, the formulation of policy responses and the role of specific actors within the policy process.^30^ Advocacy coalitions framework scholars define advocacy coalitions in terms of “deep core,” “policy core,” and “secondary” beliefs to delineate different types of belief and their relative durability and importance in shaping actors’ policy positions.^31^ Here we do not seek to differentiate between these different types of beliefs and their relative mutability. Instead, we employ the wider concept of “belief coalitions” to capture commonalities of belief between policy actors on the specific issue of alcohol policy and COI. Previous studies have identified the existence of coalitions with shared beliefs and interest relating to alcohol policy in Ireland and the United Kingdom.^32-34^ However in this study we seek to examine not specific policy outcomes that a coalition of advocates are seeking to achieve but their underlying beliefs about the alcohol policy process and legitimacy of certain policy actors which in turn shape policy-making.

 Following specific interview questions, respondents were categorised into two “belief coalitions” on the basis of their views and general orientation to alcohol related, harms, the most appropriate policy responses to this and the legitimate role of the alcohol industry in policy-making and implementation. The definitions of, and positions adopted on the concept of COI in alcohol policy by different actors, and the similarities and differences which emerged in relation to these both within and between these coalitions were then examined.^30^ The findings presented below identify the dominant coalitions which emerge and the beliefs expressed regarding COI and industry engagement in policy-making. Quotations from interviews are labelled according to membership of either the “public health” (PH) or “industry partnership” (IP) coalitions, with each respondent allocated a specific identification number (eg, PH1, IP2, etc).

## Results

###  Identifying Belief Coalitions

 Two competing coalitions were identified amongst participants: the “public health coalition” (PHC) and the IPC. The PHC included health campaigners, academic researchers and medical professionals, while the IPC included alcohol industry representatives. Two stakeholder groups—parliamentarians and civil servants—were split across the two coalitions. Membership of coalitions was determined by shared beliefs relating to the nature of alcohol harm and support for policy responses. Table 3 presents a summary of the two belief coalitions as identified by researchers on the basis of the policy positions articulated by interviewees and in industry documents.

**Table 3:** Belief Coalition Membership Among Interview Participants

**Advocacy Coalition**	**Public Health**	**Industry Partnership**
Membership	Health campaigners Academic pesearchers Medical professionals Parliamentarians Civil servants	Alcohol industry Civil servants Parliamentarians
Shared policy beliefs	Alcohol harm trends worsening Alcohol causes population-wide harms, beyond individual drinkers Evidence-based policies required to tackle harm at population-level Restrictions on price, availability and marketing most effective policy solutions	Alcohol harm trends stabilising/improving Alcohol harms concentrated in small minority of irresponsible drinkers Targeted measures required that do not affect majority of responsible drinkers Industry voluntary partnerships and self-regulatory measures are the most appropriate policy responses

 The IPC favoured voluntary partnerships with alcohol industry groups over government regulation. This was the most notable shared belief between this coalition, distinguishing from those belonging to the PHC. Industry actors within this coalition (both in interviews and documents analysed) described adverse impacts from alcohol as experienced by a small minority as opposed to a large proportion of the population and identified the problem in the United Kingdom in terms of individual health and crime indicators. In addition, they highlighted the health and social benefits of alcohol.

 Those in the PHC expressed almost uniform beliefs about the nature of alcohol harm and for the corrective policy responses to these in line with the research evidence base discussed above. This was the most notable shared belief between members of this coalition, distinguishing from those belonging to the IPC. The uniformity of responses from participants identifying with a public health approach to reducing alcohol harm was remarkable. The perceived consensus on the evidence to support a policy framework to reduce alcohol harm was noted by participants, who described alcohol policy as an unusual field of research where contestation was uncommon:

 “*So, I think the evidence there is particularly clear about what works and what doesn’t work [...] it’s not one of these issues where there’s much debate or controversy. We know that restrictions on availability are effective and cost effective, restrictions on advertising and marketing, which the industry disputes, you know the balance of evidence suggests that it is likely to be effective. So, I think, what I think is really interesting is that it’s not an area where the evidence pulls in different directions, particularly in relation to availability and pricing”* [PH1].

###  Understanding of Conflicts of Interest

 Both belief coalitions expressed a belief that COI exists in alcohol policy settings. However, when asked directly how they would describe what a COI is, many respondents struggled to articulate a clear conceptual definition of COI. Indeed, several respondents in the PHC demonstrated both amusement and embarrassment at the fact they were unable to describe something about which they held such strong positions. As a result, the descriptions of COI offered by all participants were extremely varied and many respondents appeared to question their own understanding of the concept when attempting to articulate a definition. For example, one academic researcher was not able to provide a concise definition and had to repeatedly pause and reflect on their thoughts:

 “*So, I think it might be something where [pauses] the goals [pauses]. I suppose I need to think of it in terms of a particular context, I suppose in relation to this issue, I suppose a meeting or a decision or participation in this process. So, I guess it would be where the goals of that process, or the goals of what you are doing, or whatever it is, are not [pauses] I was going to say aligned but I suppose it’s not about having an alignment but where your goals or the influences on your goals are not in alignment with the goals of that process”* [PH1].

 In contrast, industry representatives seemed to have less difficulty in defining the issue. One such respondent described COI as arising when opposing views means a mutual goal cannot be met:

 “*I guess its two people that have got an opposing view on something which will allow or force either of them to not meet whatever their objectives are, I’m sure there’s a much better definition that I’ve no doubt you have studied endlessly, but for me it’s you know, if somebody is forcing you to do something that is bad for your corporate or your personal objectives and therefore you can’t find a middle ground*” [IP1].

 The aspect of COI that both coalitions were most comfortable describing was financial: ie, being in receipt of funds from organisations whose objectives were contrary to the goals of a particular programme or activity. This was seen as representing the clearest example of COI. For example, as one health campaigner belonging to the PHC commented:

 “*Someone has a conflict of interest if they are involved in some kind of decision-making process or regulatory process, when actually they have a financial or other benefit which might influence how they would view that policy and what advice they would give in terms of what direction that policy should be taking”* [PH3].

 This was mirrored by an industry representative who cited the conflict between profit motives of alcohol companies and public health objectives:

 “*There’s undoubtedly a conflict between a lot of the alcohol policy and the views of many organisations in terms of how they think they are best positioned to return profit to their shareholders basically and they see the government as something, largely the government but also the health lobbyists, will make their job more difficult and that’s something that clearly creates a conflict because the objectives are different. One wants to reduce alcohol, one wants to sell more, and it’s clear that that conflict is obvious and easy to understand”* [IP1].

 However, respondents in both coalitions described how COI was not limited to financial transactions, and also described a range of non-financial forms of COI. These tended to focus on individual, as opposed to institutional level conflicts and included personal relationships and obligations, professional experience and membership of organisations, boards or networks. When describing these non-financial examples, participants expressed a belief that COI extends to actors within the alcohol policy subsystem beyond the alcohol industry. The descriptions of COI amongst non-industry actors included personal attributes beyond an individual’s control, for example, unconscious bias, ethnic and cultural background, professional experience, and education. One academic researcher described how the involvement of medical professionals and academics in advocacy activities was seen as increasing the risk of bias in alcohol policy research as they became committed to fixed positions:

 “*There are also, in alcohol policy a large number of academics who wear multiple hats, so there are academics who are also health professionals and therefore have a strong commitment towards improving the health of the public, in a professional sense rather than in the general academic sense of making a better society. There are also a number of academics who act as advocates, and thus in some sense commit themselves to a position, and as a result you could argue that position may influence the way they go about their research in a whole range of ways, from the questions they ask to what they promote from their findings”* [PH4].

 However, concerns were raised about equating these with the financial COI of the industry and a number of respondents in the PHC described how commercial financial COI should have a ‘special status’ compared to other actors’ interests in alcohol policy settings. One medical professional commented:

 “*If you’re an NHS doctor pushing for the expansion of your treatment field then yes you have your patients in mind but it’s also pretty good for your career, you know, getting investment and getting a bigger clinical team, you get a bigger research unit and so on […]. But my own view is that I think it’s appropriate that the financial and corporate conflict of interest is seen as having a particular importance and a particular relevance here, so there are those conflicts right through advocacy work that there will be various bits of self-interest, but I think in the actual real world of alcohol policy and you look at the history of that, financial and corporate conflicts of interest I think have a particular status and a particular importance”* [PH5].

###  Conflicts of Interest and Alcohol Industry Engagement in Policy

 COI and alcohol industry involvement in alcohol policy were described by both coalitions as variable and context specific, depending on the type of industry actor, the stage of policy process, and the type of engagement activity. There was little variation in participant beliefs within coalitions. The two competing coalitions had both divergent and convergent beliefs relating to alcohol industry engagement in policy.

 Participants were asked to comment on whether they believed the level of COI, or any associated risks to public health outcomes, varied according to the *type of alcohol industry actor* that was engaged in the policy process. A variety of actors were described, including retailers (both on- and off-trade), producers (of different types of alcoholic beverages and multinational and domestic), trade associations, Social Aspects/Public Relations Organisations (SAPROs) and NGOs, think tanks and individual experts in receipt of industry funding.

 On-trade retailers were described by participants as presenting a “lower” level of COI compared to off-trade retailers, and multinational producers were described as more ‘highly conflicted’ than domestic or artisanal producers. For example, one industry representative described how small, domestic and artisan producers were more socially responsible and connected with their consumers compared to large, multinational producers:

 “*There’s been a lot of growth of small craft brewers. And those guys have got a very close relationship with their nearby consumer groups, they probably know by name their best customers and they are sourcing products from the farm next door and selling it down the road. So they’ve clearly got a very different perspective to the big multinational companies, the AB InBev, the Heinekens and Diageos and so on, who have board meetings in cities around the world, in fact on different continents, and will have a different perspective*” [IP1].

 Multinational alcohol producers were described by participants as dominating alcohol trade associations and, therefore, wielding greater influence over UK alcohol policy than smaller domestic producers and retailers. A lack of progress in promoting the UK Chief Medical Officers’ low risk drinking guidelines was attributed to the influence of multinational producers within trade bodies who had rejected calls to place the updated guidelines on alcohol product labels. Similarly, participants described how support for MUP amongst on-trade retailers, who would be largely unaffected by the measure, was reportedly hampered by representations from multinational brewers within pub trade associations:

 “*The pubs have a very good case for being given support, they are social hubs and community centres and I personally regret that they are disappearing at the pace that they are. The drinks they sell are all way above the minimum unit price and we seek to try and persuade their representatives that they have an interest in pushing for MUP but they then turn back to the BBPA, their trade association, which is strongly against MUP […] But the reality is, they also represent the brewers, who produce the beer that is then canned and sold in the supermarkets, so there is a conflict of interest within that organization”* [PH7].

 Those in the IPC were more likely to describe differences in COI associated with different types of beverages. However, the PHC did not see any variance in level of COI in terms of producers of different beverage types.

 Within the PHC, the level of COI associated with NGOs and individual experts in receipt of industry funding varied according to type of organisation and funding relationship. Industry-funded SAPROs such as Drinkaware and pro-business think tanks such as the Institute for Economic Affairs were described as presenting more insidious COI than alcohol companies and/or trade associations. Their operations were described as covert and more deceptive because of their claims to legitimate engagement in public health policy development and implementation. For example, a health campaigner commented:

 “*I think the approach to something like Drinkaware is more insidious, it’s in many ways more dangerous because actually its whole remit of operations, no matter how well you think it is organised in how it carries out that remit, is designed to focus on an area of activity in alcohol harm which is one of the least effective areas […] and I say insidious because it describes itself as an independent charity. Well to me it cannot possibly be independent if 90 per cent of its funding comes from the alcohol industry” *[PH3].

 However, NGOs that received modest sums of money from alcohol industry bodies (but did not rely on industry funds to exist) and whose organisational objectives and activities were perceived as in the public interest were considered as presenting less of a conflict. One researcher commented on the difference between SAPRO Drinkaware and the treatment provider formerly known as Addaction:

 “*Clearly Drinkaware exist to act, to essentially ensure that the government is not leading the campaign against alcohol in terms of social marketing – it’s there to provide a kind of shield for the industry. Addaction don’t exist for that reason, they exist for their own reasons and they have their own motivations, and while they may have a conflict of interest because of their industry funding, that doesn’t necessarily mean you should not touch them with a barge pole as a result”* [PH4].

 Agreement was evident across both belief coalitions with respect to the scale of COI associated with alcohol industry engagement at some of the *different stages of the policy process*^[1]^.^35^ the policy adoption and implementation stages were described as opportunities for industry to inform and enact policies by drawing on their technical expertise and capabilities. Responding to public consultations and recorded formal meetings with officials and ministers, that were a matter of public record, were seen as examples of how industry could inform the finer details of policies. However, divergent beliefs existed between the two coalitions about industry’s role in the agenda setting/problem identification and evaluation stages of the policy process. PHC members reported that industry influence during the problem identification stage could frame the policy debate in terms which would narrow the scope for evidence-based policy solutions to emerge and promote substitute policies such as voluntary partnerships which have limited impact on health outcomes. One researcher commented:

 “*I think the risk is highest at the start because at the start you have industry framing the problem in particular ways that they can be quite effective at […] So if you frame a problem in terms of individual responsibility and you misrepresent the evidence, and that has been your job for 20 years, it’s very difficult for someone outside of that process or someone who’s very new to that process, or even for someone for whom it isn’t their day to day work, to see that it is a problem that is being framed in a certain way”* [PH1].

 Members of the IPC acknowledged that involving the alcohol industry at the early stages of the policy process could limit policy selection. A civil servant described how, when presented with all stakeholder views on policy options, ministers might be less inclined to pursue certain ideas if they were made aware of industry opposition. However, this was seen as vital to policy progress, because ministers needed to be made aware of all arguments to judge whether a policy would survive the parliamentary process:

 “*Within the general ebb and flow of policy, it’s the policy official’s job to be able to talk ministers through ‘well if you do this, then this is likely to happen and these people will say this’, and so in a way, you’ve kind of got to have some of those discussions, because otherwise you’re not going to be able to prepare your minister for what happens. And in a way, then it kind of tempers their realism, or tempers their ambitions”* [IP2].

 Support amongst both coalitions was identified for certain *types of industry engagement*. Implementation of server training schemes for licensed retail staff and product reformulation to reduce alcohol by volume % strength of beverages are two examples. Both coalitions also shared beliefs regarding opposition to alcohol industry involvement in pricing policies. Setting the level for MUP and developing alcohol duty structures was referenced by several participants as a “red line” where industry must not be involved:

 “*Where tax is involved, again, they can lobby but you’ve got to be careful about having anyone from the industry sat on any groups in the Treasury or elsewhere that has an undue influence on the setting of that tax or anything like that”* [IP3].

 Both coalitions also described similar beliefs relating to industry undeclared funding for research reports. Not declaring that industry had funded research reports which were used to influence decision-makers’ views on policy issues was labelled corrupt by one PHC member:

 “*What I think is corrupt is not too strong a word is to say ‘we will go to a supposed independent think tank or academic and actually because we’re giving them money, they will help produce things that are favourable to our case which they will then publicise. And they will be used by supposedly independent, reputable organisations like the BBC as experts in the area’” *[PH7].

 Corporate hospitality outside of parliament, such as invitations to sporting events or drinks parties, was described by members of both coalitions as presenting risks linked to COI. Civil servants described how they often received such invitations, however they reported declining due to a view that attending social events was not appropriate. Similarly, one civil servant from the IPC described how it should be unacceptable for parliamentarians to receive corporate hospitality:

 “*I think extending hospitality to, tickets to Wimbledon or whatever, to MPs, that is a direct conflict of interest. That should be on the register of interest and declared, and it should be culturally unacceptable for members of parliament or policymakers to accept that kind of hospitality. That’s direct, it’s indirect in that it’s hospitality, but they are accepting a gift in kind from alcohol producers and I think that is a very significant conflict of interest” *[IP2].

 Whilst most members of the PHC declared they would not be willing to accept alcohol industry funding as a means of protecting against COI, several participants would consider other non-financial forms of engagement if certain conditions were in place. Sharing a public platform, attending events and roundtable meetings where industry was present was described as a “grey area” for health campaigners, who preferred to judge on a case-by-case basis whether the risk of COI was too great. One health campaigner described that they would not engage in bilateral discussions with the alcohol industry, however if meetings were convened by a government body, they would be willing to engage:

 “*We only engage in mechanisms that are tripartite, where there is the NGOs, the academia and the government and those are the only fora where if we have to engage with industry, we will be there as well. And that’s because we recognise that at some point the industry needs to speak formally with government”* [PH6].

 Transparency was considered a key factor in managing COI and protecting public policies from undue influence. Both coalitions described how unrecorded, informal engagements with decision-makers were undesirable and could lead to deleterious outcomes. Formal engagements in the policy process that were a matter of public record such as meetings with officials and entertainment within parliament could be considered as acceptable forms of alcohol industry engagement by the PHC. However, whilst declarations of interest were described as important, they were not described as tools that would eliminate or reduce COI. Rather, they were described as instruments to manage COI and mitigate risks associated with covert influence.

###  Cross Industry Comparisons

 Similarities in tactics between the alcohol industry and other unhealthy commodity industries – most notably tobacco and foods high in fat, salt and sugar – were identified. However, despite the similarities described by some participants regarding the industry’s corporate political activities, the majority of interviewees, from both coalitions, did not think that the alcohol industry should be subjected to the same restrictions as the tobacco industry, which is excluded from many aspects of the public policy process under Article 5.3 of the WHO Framework Convention on Tobacco Control (FCTC).^13^ Alcohol industry representatives strongly resisted comparisons between tobacco and alcohol products. One participant commented:

 “*I think that tobacco is totally different from the brewing industry. Tobacco is bad for you, full stop. If you smoke, it will cause you problems. Whereas I believe that drinking in moderation can be absolutely fine, I think it can be good for your mental health and wellbeing, and in the social situation where you drink it, there is some evidence, and I certainly wouldn’t want to go into some medical publication, but there is quite a lot of good evidence around cardiovascular and around various other things to do with drinking in moderation and that was never true of tobacco, so to me the two are really very different*” [IP3].

 PHC members also described differences between the two products and the UK Government’s long-term aim to eradicate smoking was cited by respondents as a reason to treat the two products and thus the two industries differently. It was acknowledged by both coalitions that the majority of people in the United Kingdom enjoy alcohol, which is seen as a normal part of everyday life, therefore the alcohol industry should not be excluded from society in the same way the tobacco industry has been. For example, one medical professional commented:

 “*So what we haven’t discussed is the right of the drinks industry to exist. And they do have a right to exist, you know, the majority of the people in this country like to have a drink. And also a majority of people gain a lot of enjoyment from having a drink. So, I think it’s very important just to sort of bear that in mind as well. It’s difficult to make the same statements about the tobacco industry. But the drinks industry, they have a right to exist”* [PH5].

## Discussion

 This exploration of different aspects of COI, as perceived by UK alcohol policy actors, helps to deepen our understanding of this concept, and will thus inform efforts to develop operationalizable definitions of COI, and guidelines for practice, in alcohol policy settings. The difficulty experienced by many respondents in defining COI speaks to the need for more conscious engagement with this issue by policy actors and a reflexive engagement on their practice related to these issues. This may facilitate both more effective policy responses and a less febrile policy discourse. The aspect of COI that participants were most comfortable in describing was financial COI and both coalitions identified that alcohol industry funding of health-related activities represented a clear financial COI. This is in keeping with debates within the existing literature on the distinction between financial and non-financial COI and that financial COI are more easily identifiable, measurable and thus amenable to disclosure and mitigation.^16-20,36^ However, this study identified shared beliefs that protecting against financial COI alone would not sufficiently guard public policy against undue influence from the alcohol industry. Participants’ understanding of COI included a broad range of non-financial forms, which tended to focus on individual, as opposed to institutional, level conflicts. However, a number of respondents in the PHC described how financial COI on the part of commercial actors should be awarded “special status” compared to other actors’ interests in alcohol policy settings. This theme is explored by Bero and Grundy who argue that conflation of “COI” with “interests” in general serves to muddy the waters about how to manage COI in relation to research settings.^20^ They argue that, whilst it is essential to systematically examine all social values shaping a research process, these cannot be eliminated and must instead be made visible and open to critical interpretation.^20^ This process could be applied to alcohol policy settings to ensure that actors’ interests in policy outcomes – both financial and non-financial – are transparently presented so they can be assessed according to their conflict with public policy goals.

 The range of different alcohol industry actors described by respondents in both coalitions demonstrates their understanding of the diverse nature of the alcohol industry. Attempting to define the alcohol industry and sub-categories of actors enabled participants to reflect on the COI presented by different sectors of the industry. This differentiated approach to the alcohol industry could allow for the development of more nuanced guidelines for engaging with different types of industry actor.

 PHC members expressed concerns about informal versus formal engagement between industry and decision-makers, especially at the agenda setting stage of the policy cycle. Unrecorded meetings with policy officials and corporate hospitality outside parliament were perceived by members of both coalitions as presenting higher risk of COI than recorded meetings and entertainment within parliament. Transparency in relation to industry engagement in the policy process was described by participants as a key tool for managing and mitigating risks associated with COI, which could be explored further via aforementioned guidelines.

 An important finding from this study relates to how those in the PHC perceive the COI presented by the alcohol industry in comparison to the tobacco industry. Calls have been made from international public health researchers and campaign groups to apply the same restrictions on alcohol industry engagement in public policy as is currently applied to the tobacco industry under Article 5.3 of the WHO FCTC.^10,37^ However, little support was identified amongst both coalitions for treating the alcohol industry the same as tobacco companies. There was an acknowledgement that public health goals for tobacco control and alcohol harm reduction were divergent in their end games: the UK aspired towards a smoke-free society, but no public health groups sought complete alcohol prohibition. The alcohol industry was perceived as a legitimate actor within UK society with a “right” to exist and, therefore, a “right” to be consulted by government on issues that affect their business. It can be argued that respondents in both coalitions held beliefs relating to democracy and the importance of stakeholder consultation in good governance processes, which took precedence over beliefs relating to the risk of alcohol industry COI in health policy decisions.

 Previous studies have assessed how alcohol industry activities have impacted on policy outcomes, and how the alcohol research community views alcohol industry involvement in science.^38^ Others have addressed similar issues in cross-industry, international comparative perspective.^21^ However, no investigations have been conducted to assess the views of UK policy actors, across sectors, on COI in alcohol policy. This study, therefore, makes an important contribution to a growing body of evidence about alcohol industry involvement in public health.

 This study finds evidence to suggest policy actors from “public health” and “industry partnership” belief coalitions would support the development of guidelines designed to manage COI in UK alcohol policy settings which introduced greater transparency about alcohol industry involvement in policy processes. Calls for greater transparency align with international guidelines for governmental institutions on engaging with non-state actors: The WHO handbook for engaging with non-state actors mandates that all WHO interaction with non-State actors must be managed transparently,^39^ and the US Centre for Disease Control guiding principles for public-private partnerships identifies transparency as a core principle for effective communication with the private sector.^40^

 Finally, there are methodological issues raised by the analysis presented above which are of wider relevance. The study employs interviews with industry actors which have been used previously to study UK alcohol policy.^4,33^ However, subsequent analyses have eschewed industry respondents.^41,42^ While interviews with industry actors have informed studies of other health harming industries (eg, gambling) they are not deemed to be acceptable in the field of tobacco control, due largely to the expansive interpretation of norms of non-engagement within the WHO FCTC.^43^ As such, there are potential risks associated with industry engagement in terms of becoming a conduit for industry messaging and the perception of the independence and ethics of the study by public health actors and researchers. Despite this the advantages of including industry perspectives outweigh the potential disadvantages. Given the focus of the present study on issues of COI as they explicitly relate to industry actors it is imperative to include these actors in the study to capture the arguments that these actors make to legitimate their involvement in policy-making. Moreover, it could be interpreted that the authors had already ‘taken sides’ in the debate about industry involvement in policy debates had they excluded these actors from their study before the fact.

 While the focus of the study is on the UK context, the issues addressed and the potential findings are widely applicable given the similar lack of engagement with COI and the alcohol industry in other national settings, and the lack of global norms, standards and guidelines in this area identified in the introduction.

###  Strengths and Limitations of Study

 This article explores and presents novel data on an underexplored, yet highly contentious topic, with important implications for the development of effective health policy. Additional strengths include the breadth of policy actors who participated in interviews, and the inclusion of alcohol industry and decision-maker perspectives, stakeholders who are often difficult for public health research to access.

 The relatively low number of industry respondents recruited means these were supplemented through industry consultation responses which do not focus on the specific research questions examined in the study or allow the probing and follow up to responses possible within an interview. Nevertheless, they provide a wider articulation of industry positions than the two interviews that were conducted and so are a valuable data source.

## Conclusions

 This study presents findings which enhance our knowledge about how UK alcohol policy actors understand the concept of COI in relation to alcohol industry engagement in policy processes. In doing so, it underlines again that the UK alcohol policy subsystem is a complex and contested space. However, policy actors exhibit more nuanced understandings of COI than is often recognised in scholarly literature or is immediately evident in policy debates, which often present the issue as a binary choice between engagement and non-engagement with alcohol industry bodies. Highlighting this may enable the development of shared understandings of COI that can form the basis for alcohol-specific guidelines to inform policy-making and practice similar to those which exist in other areas of health policy. Findings from this study may also inform refinement of existing frameworks and the development of new guidelines in other areas of public health policy where the impact of economic actors is becoming increasingly recognised, such as gambling.

## Ethical issues

 This study was approved by the London School of Hygiene and Tropical Medicine.

## Competing interests

 Authors declare that they have no competing interests.

## Funding

 Katherine Severi is employed by the Institute of Alcohol Studies (IAS). IAS receives funding from the Alliance House Foundation. Benjamin Hawkins’ position is supported by the Medical Research Council [grant number: MC_UU_00006/7].

## Endnotes

[1] The policy stages framework used for this study consisted of five stages: (1) agenda setting/problem identification, (2) policy formulation, (3) policy adoption, (4) implementation, (5) evaluation (See Lasswell^35^).
